# Correlations between Aging, Telomeres, and Natural Compounds

**DOI:** 10.14336/AD.2025.0853

**Published:** 2026-09-01

**Authors:** Katarzyna Rakoczy, Laura Wojdyło, Sara Suwała, Karolina Klasen, Jacek Kuźnicki, Marek Ziętek, Julita Kulbacka

**Affiliations:** ^1^Faculty of Medicine, Wroclaw Medical University, Wroclaw, Poland.; ^2^Department of Experimental Dentistry, Wroclaw Medical University, Poland.; ^3^Department of Molecular and Cellular Biology, Faculty of Pharmacy, Wroclaw Medical University, Wroclaw, Poland.; ^4^Department of Immunology and Bioelectrochemistry, State Research Institute Centre for Innovative Medicine, Vilnius, Lithuania.

**Keywords:** aging, telomeres, natural compounds, flavonoids, vitamins

## Abstract

Aging constitutes a complex biological phenomenon that is shaped and modulated by a wide range of genetic, metabolic, and environmental factors. A key characteristic of aging is the progressive shortening of telomeres, a process worsened by both physical and mental stressors. This shortening is interdependent with oxidative imbalance, persistent low-level inflammation, mitochondrial decline, and defective DNA repair mechanisms, factors that collectively drive age-associated disorders. Our review highlights the role of telomeres in the aging process. It examines the therapeutic potential of varied natural compounds, including flavonoids, carotenoids, peptides, polysaccharides, essential oils, and postbiotics in modulating these age-associated pathways. Experimental findings suggest that such compounds may safeguard telomeres, diminish oxidative injury, stabilize inflammatory signaling, and support mitochondrial bioenergetics. The convergence of findings from molecular, cellular, and *in vivo* studies supports the notion that integrating such compounds into both therapeutic and preventive dietary strategies may not only contribute to prolonged lifespan but also significantly enhance healthspan, the portion of life spent in good physical and cognitive health. However, the complex nature of aging pathways means that these promising insights also emphasize the importance of multidisciplinary research, which is indispensable for validating efficacy in clinical settings and exploring synergistic effects. Thorough human studies evaluating dosage, bioavailability, long-term safety, and population-specific effects are necessary to translate encouraging in vitro and animal findings into safe and effective interventions. The integrated examination of telomere biology and six classes of natural compounds along three main mechanistic axes—mitochondrial function, inflammatory modulation, and telomere maintenance—with a focus on translational relevance, is what makes this review novel. The dynamic relationship between telomere biology and naturally derived bioactive substances offers an interesting direction for low-risk interventions aimed at promoting healthy aging and reducing the global burden of age-related diseases.

## 1. Introduction

Aging is marked by a gradual decline in the body’s ability to maintain tissue structure and function. It results from many cellular and biochemical processes, including telomere shortening, which accelerates under both physical and psychological stress [1, 2]. Another hallmark of aging is persistent, low-level inflammation, often referred to as ‘inflammaging’, which some studies regard as a powerful predictor of lifespan [3]. Telomere shortening can itself promote a pro-inflammatory phenotype in cells, as critically short telomeres trigger DNA damage responses that upregulate inflammatory mediators. Inflammation is associated with age-related sarcopenia. A recent study identified six cytokines: hepatocyte growth factor (HGF), interferon gamma (IFNγ)-induced protein 10 (IP-10) (also known as CXCL-10), macrophage colony-stimulating factor (M-CSF), interleukin-7 (IL-7), and monocyte chemotactic protein 3 (MCP-3). RANTES (CCL5) is upregulated upon T-cell activation; its circulating levels change in sarcopenia and may exert protective effects [4]. Among other factors that contribute to aging, immune system decline, oxidative stress, and genomic replication errors can be enumerated, which are often influenced by genetic predispositions and lifestyle choices. Multiple cellular stressors contribute to aging. These include ultraviolet radiation, as well as depletion of vital coenzymes such as nicotinamide adenine dinucleotide (NAD) [5], a decline in key antioxidants such as glutathione (GSH) and the superoxide dismutase (SOD) [6], mitochondrial dysfunction [7], and genetic mutations [8] converge to drive the onset and progression of age-related diseases, significantly undermining human health over time. As telomeric DNA is particularly sensitive to oxidative damage, excessive ROS can accelerate its erosion, reinforcing a self-perpetuating cycle of redox imbalance, chronic inflammation, and progressive telomere shortening during aging [5-8].

Natural compounds like dietary polyphenols possess a range of biological activities, including anti-inflammatory effects, free radical neutralization, neuroprotective benefits, and anti-aging properties, which encourage the incorporation of these compounds into holistic anti-aging therapeutic approaches [9]. Free radicals constitute unstable molecules that are the foundation of oxidative stress and directly lead to inflammation and cellular damage, thus contributing to the process of aging. Natural compounds, acting as scavengers, protect cells from oxidative stress and, in this way, protect the organism from age-related diseases, including cancer [10], cardiovascular diseases [11], and neurodegenerative diseases (NDs) [12]. In addition to their antioxidant capabilities, polyphenols can modulate immune function, regulate inflammatory responses, and enhance resistance to diseases such as systemic lupus erythematosus [13], rheumatoid arthritis [14], and multiple sclerosis [15]. Described modulation is based on inhibiting toll-like receptors (TLRs) as well as NF-κB and MAPK/ERK pathways, regulating pro-inflammatory gene expression, suppressing the enzymes associated with the reactive oxygen species (ROS) production like xanthine oxidase, and up-regulating antioxidant enzymes, such as SOD, catalase, and GSH peroxidase (GPx) [16]. The neuroprotective potential of natural compounds is based on their ability to enhance mitochondrial function and support DNA repair pathways. Although the brain metabolic rate of polyphenols is relatively low, studies have shown that flavonoids can reduce oxidative stress and neuroinflammation, thereby slowing brain aging [17]

The risk of various metabolic disorders increases as the aging process progresses. Natural compounds, such as polyphenols, can influence various metabolic processes, such as the regulation of blood glucose and lipid levels [18]. For instance, strawberry juice, rich in polyphenols, demonstrates hypoglycemic and hypolipidemic effects in streptozotocin-induced diabetic rats, effectively restoring carbohydrate metabolism enzymes and antioxidant enzyme activities to normal levels while significantly reducing lipid peroxidation and proinflammatory cytokines [19]. Dietary polyphenols could therefore be incorporated into the pharmacological regulation of glucose and lipid metabolism, which is commonly disrupted as the aging process proceeds, contributing to the occurrence of age-related diseases. Studies indicated that an intake of ≥550 mg of flavonoids per day significantly reduces the risk of type 2 diabetes mellitus (DM) [20].

Telomere shortening can cause inflammation, inflammation increases oxidative stress, and oxidative stress speeds up telomere erosion. These three factors are closely related to telomere attrition, chronic inflammation, and redox imbalance. Many age-related functional declines are caused by a feedback loop that they all contribute to. A unified framework for assessing interventions targeted at healthy aging is provided by an understanding of this interaction.

### 1.1 The hallmarks of aging

The definition of aging remains debated: some interpret it as a normal biological progression, whereas others consider it largely pathological, reflecting the accumulation of stressors and cellular damage throughout life [21, 22]. Regardless of definition, at the molecular and cellular levels, aging results from several interconnected mechanisms *(Table 1)* and is the primary risk factor for most chronic diseases. As the immune system becomes less reliable and efficient with age, the elderly face a heightened risk of chronic inflammation-related conditions such as neoplastic diseases, cardiovascular diseases, and an increased vulnerability to a variety of infectious diseases [23]. As global life expectancy increases, so does the burden of age-related diseases. Consequently, it is of great importance to understand and mitigate the mechanisms of aging, aiming not only to extend lifespan but also to enhance healthspan—the period of life spent in good health. By addressing the underlying biological processes that drive aging, it might be possible to delay or prevent age-related diseases, improving overall well-being and reducing healthcare costs [24, 25]. Geroprotectors constitute the agents that pharmacologically slow aging in model animals. While hundreds of compounds have been shown to extend lifespan in laboratory models, clinical studies on potential geroprotectors, particularly in healthy older adults, remain extremely limited. Based on their functions, the mentioned compounds can be grouped into categories such as antioxidants, proteostasis regulators, genomic instability suppressors, epigenetic modulators, mitochondrial function preservers, inhibitors of aging-related signaling pathways, senolytics and senostatics, anti-inflammatory agents, antifibrotic compounds, as well as prebiotics and enterosorbents [26]. Importantly, potential geroprotectors include not only synthetic compounds but also substances derived from food or produced by symbiotic gut microbiota, such as spermidine and vitamin K2 [27].

**Table 1:** Cellular and molecular mechanisms contributing to the aging process.

Hallmarks of aging	Age-related changes	References
***Genetic factors***		
**Nuclear DNA damage**	Genome instability, accumulation of genetic damage, impaired DNA repair system, and accumulation of aneuploidy in the brain	[28-30]
**Epigenetic alterations**	•DNA methylation signatures, often referred to as epigenetic clocks, offer a reproducible biomarker of biological age by examining methylation patterns at selected genomic lociSIRT6 loss leads to abnormalities in mice that overlap with aging-associated degenerative processes. •The loss of heterochromatin is proposed as a key contributor to the aging process. Heterochromatin formation has been shown to extend lifespan and regulate ribosomal RNA (rRNA) synthesis in *Drosophila*. Reduced levels of heterochromatin lead to a significant decrease in lifespan, while increased heterochromatin enhances longevity. These lifespan changes are associated with improved muscle integrity. As organisms age, heterochromatin levels naturally decline, and its formation plays a crucial role in silencing rRNA transcription. Preserving genome stability, particularly at the ribosomal DNA (rDNA) locus, and inhibiting unnecessary rRNA production appear to be mechanisms promoting lifespan.	[31-33]
**Mitochondrial DNA haplogroup**	Research highlights associations between specific mtDNA haplogroups and human longevity. For instance, the C150T polymorphism has been linked to extended lifespans in Finnish and Japanese populations. Haplogroup J is notably over-represented in long-lived populations, including centenarians from northern Italy, Ireland, and Finland, but underrepresented in Chinese Uyghur nonagenarians, which suggests a connection between mtDNA haplogroups and aging. However, the effects appear to vary by population.	[34-41]
**Mitochondrial DNA damage**	There is a strong correlation between mtDNA damage and aging. Mitochondrial dysfunction in aging is largely linked to the clonal spread of mutations that arise in mtDNA early in development and later accumulate within cells. While the overall frequency of mtDNA mutations remains low, the accumulation within individual aging cells can become significant. Over time, this may lead to homoplasmy, a state where mutant mtDNA predominates over the normal genome within a cell.	[28, 42-44]
**Telomere attrition**	Telomere length is widely considered a molecular indicator of cellular age. Health-promoting behaviors are associated with longer telomeres and reduced oxidative stress, suggesting a protective effect on biological aging. Conversely, unhealthy habits correlate with shorter telomeres and accelerated aging processes.	[45]
***Proteostasis***		
**Chaperone-mediated protein folding**	A better ability to cope with various stresses, largely mediated by chaperones, significantly contributes to lifespan extension. Chaperones play a critical role in maintaining protein homeostasis, helping cells manage stressors like oxidative damage and misfolded proteins, which are key factors in aging. Enhanced stress resilience, facilitated by these proteins, promotes cellular function and longevity, supporting the idea that improving proteostasis can extend lifespan.	[46, 47]
**Autophagy dysfunction**	With age, the rate of intracellular protein degradation declines in various tissues and organs. This reduction in protein turnover contributes to the accumulation of damaged or misfolded proteins, a hallmark of aging that can impair cellular function and lead to age-related diseases. Autophagy plays an essential role in clearing senescent cells throughout an organism's life across all tissues. Many approaches aimed at mitigating age-related diseases converge on promoting autophagy, activating AMP kinase (AMPK) and the Sirtuin pathway, while inhibiting the mechanistic target of rapamycin (mTOR). These interventions include lifestyle changes such as caloric restriction, as well as pharmacological treatments. Drugs like rapamycin (the first FDA-approved longevity drug), along with compounds like metformin, aspirin, and resveratrol, have all shown potential in extending lifespan by modulating these pathways.	[46, 48]
**The ubiquitin-proteasome system capacity**	The capacity of the ubiquitin-proteasome system plays a critical role in determining the replicative lifespan of yeast, which suggests that enhancing proteasome function may offer beneficial effects on longevity and age-related diseases in humans.	[49]
***Cellular Senescence***		
**The INK4a/ARF locus**	The INK4a/ARF locus encodes 2 tumor suppressor molecules: p16INK4a and ARF, which constitute major mediators of cellular senescence. INK4a/ARF expression increases aging.	[50]
**Changes in composition and functions of immune cells**	Hematopoiesis declines with age, which results in a diminished production of adaptive immune cells. Regarding the nervous system, the balance of innate and adaptive immune cells in the central nervous system is modified, as compositional changes are further amplified by the weakening of the blood-brain barrier and blood-cerebrospinal fluid barrier. Myeloid cells undergo notable functional and cellular shifts with age, particularly with an increase in prostaglandin E2 signaling, which promotes pro-inflammatory pathways. While the overall number and distribution of B and T cells in the cortex remain largely unchanged with age, CD8+ T cells accumulate in the subventricular zone of the lateral ventricles, where they hinder neurogenesis through elevated IFN-γ signaling. T cell populations also increase near the sinuses, in perivascular regions, and in the hippocampus of older animals. Additionally, an expansion of natural killer (NK) cells has been observed in the hippocampus of both aged mice and humans.	[51-57]
**Inflammation**	Aging-related conditions like arteriosclerosis and neuroinflammation increase inflammatory cytokines such as IL-6 and C-reactive protein (CRP). These, combined with declining immune function and shifts in T cells towards pro-inflammatory phenotypes, lead to defective immune surveillance and systemic inflammation. Thymic involution reduces the T-cell repertoire, and the accumulation of extracellular debris and pathogens further contributes to chronic inflammation. Chronic inflammation is linked to other aging hallmarks, including genomic instability and dysfunctional autophagy. Targeting inflammatory pathways has shown promise in reversing aging-related traits. For instance, activation of the Nod-like receptor 3 (NLRP3) inflammasome leads to increased production of IL-1β, TNF, and interferons; thus, NLRP3 inflammasome knockout extends lifespan. In the nervous system, inflammatory stimuli increase the expression of vascular cell adhesion molecule 1 (VCAM1), which facilitates leukocyte interaction with brain endothelial cells and their migration into the brain. Targeting VCAM1 with antibodies or genetically eliminating *Vcam1* in brain endothelial cells reduces microglial activation and improves cognitive function in aged mice. Additionally, acid sphingomyelinase (ASM), an enzyme produced by endothelial cells, the concentrations of which rise with age, plays a role in brain aging. Reducing ASM levels in mice through genetic approaches resulted in enhanced hippocampal synaptic spine density and improved cognition.	[58-63]

### 1.2 Telomeres and telomerase

Telomeres are specialized DNA-protein structures positioned at chromosome ends. They consist of tandem repeats of the hexanucleotide sequence (TTAGGG)n, which is remarkably conserved across species [64] and protects DNA in two ways. Firstly, they act as protectors against improper DNA repair, preventing unwanted recombination and fusion events between chromosomes. Secondly, they avoid the loss of genes near the ends of chromosomes, which could otherwise be degraded due to incomplete DNA replication [65, 66]. Commonly, telomeres are progressively shortened with each cycle of DNA replication and cell division [64]. Therefore, telomere length serves as an indicator of cellular aging.

Progressive erosion of telomeres eventually crosses a critical threshold, initiating DNA damage responses that drive cells into senescence. This process prevents cell division, signaling that the cell has reached its replicative limit. It acts as a critical defense mechanism against tumorigenesis by preventing the uncontrolled proliferation of damaged cells. Without a mechanism to lengthen them, continually shortened telomeres limit the ability of cells to proliferate, which contributes to the aging process in organisms [67]. However, cells with unlimited proliferation potential, such as stem cells and cancer cells, must activate a telomere lengthening mechanism to maintain their capacity for division [68]. Cells can preserve telomere length through several pathways: reactivation of telomerase, engagement of recombination-based mechanisms termed alternative lengthening of telomeres (ALT), or more recently described processes such as intercellular telomere transfer in immune interactions [69-71]. Most malignant cells (estimated 85-95%) rely on telomerase reactivation to sustain unlimited division, whereas a smaller fraction (5-15%) maintains telomeres through ALT-mediated recombination [72]. Hence, the development of therapeutic strategies targeting telomere maintenance, such as telomerase inhibitors, is of great importance [66]. The third mechanism has recently been discovered. When an antigen-presenting cell interacts with a T-lymphocyte, it degrades the shelterin complex. It severs the telomeres with the help of the telomeric zinc-finger associated protein (TZAP) factor. The telomeres, along with Rad51, which is crucial for recombination, are then packaged into vesicles and transferred to the T-lymphocyte via the immunological synapse. This process results in an average extension of the T-lymphocytes' telomeres by 3,000 base pairs, while the presenting cell’s telomeres become shortened. The application of direct telomere transfer in medicine could offer new possibilities for addressing immune system aging, enhancing vaccination efficacy, and potentially advancing cellular rejuvenation techniques [70, 71]. When it comes to direct, macro-scale connections, Lifestyle factors that lower oxidative stress, such as regular exercise and balanced nutrition, are consistently associated with preserved telomere length and slower biological aging. In comparison, unhealthy behaviors are associated with shorter telomeres and accelerated aging [45]. Importantly, there is a direct connection between telomere status and the activation of immune antiviral defenses. Telomere shortening does, therefore, not only reflect the proliferative status of the cell, but constitutes a part of a complex mechanism that plays a crucial role in determining overall health, with its significance increasing with age [73].

**Figure 1.**
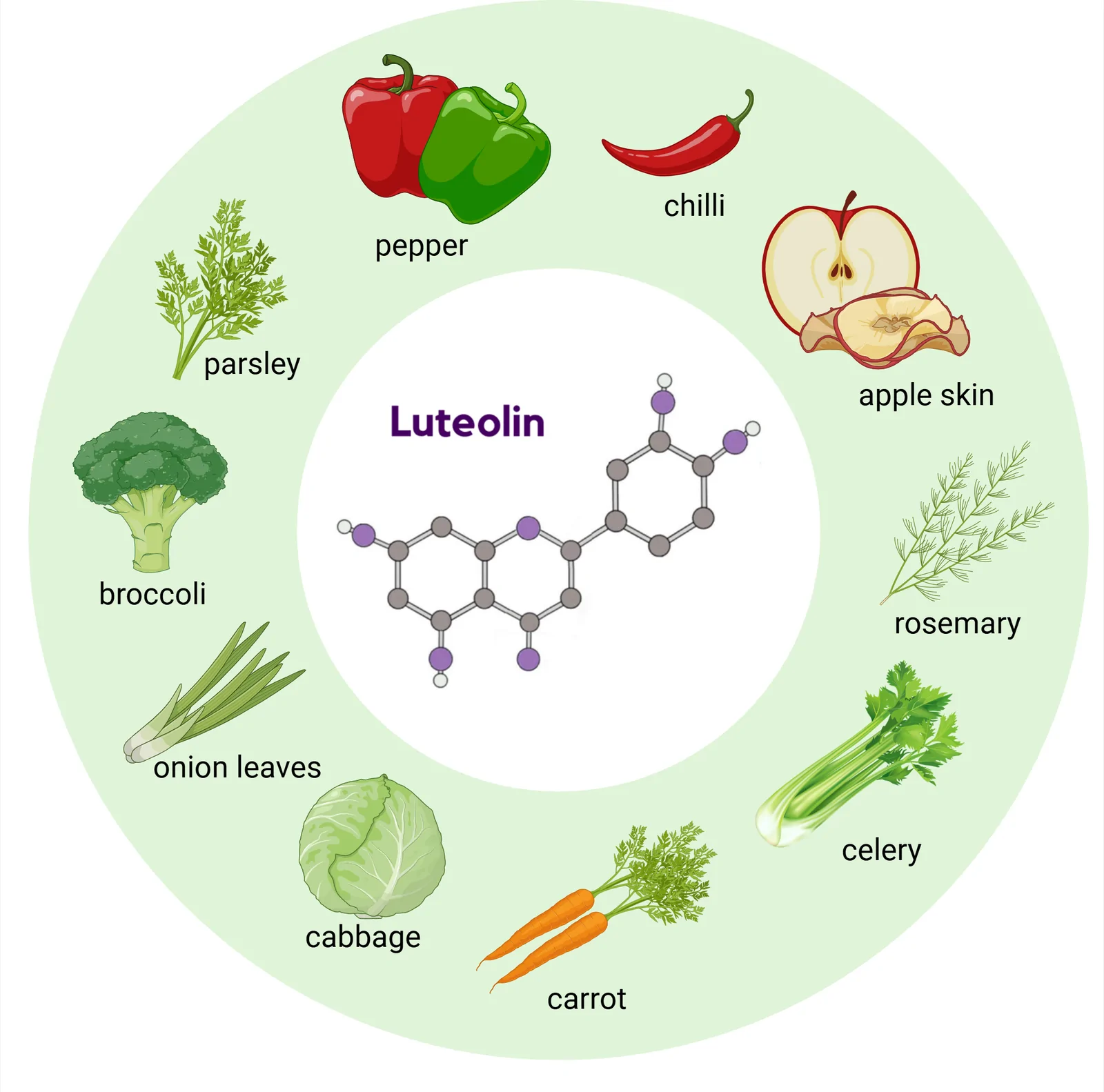
**Chemical structure of luteolin, the representative of flavones [77-80]**. Created in BioRender. Kulbacka, J. (2025) https://BioRender.com/mz0mtsm

## 2. Natural compounds

The synthesis presented here is framed by three mechanisms that are common to all the compound classes covered in this review: enhancing mitochondrial bioenergetics and redox homeostasis through pathways like SIRT1 or SIRT3 and AMPK; suppressing pro-inflammatory signaling, including NF-κB and related cytokine cascades; and supporting telomere integrity and telomerase regulation. Subsections concentrate on the subtleties unique to a compound. Together, these processes reduce oxidative damage, enhance mitochondrial efficiency, and alter the inflammatory environment, all of which are common characteristics of natural compound classes.

### 2.1 Flavonoids

Flavonoids are a diverse family of polyphenolic compounds found in fruits, vegetables, grains, tea, wine, and other plant-derived foods [74]. Beyond their role as pigments that contribute to plant coloration, more than 9,000 flavonoid structures have been described, many of which exhibit biological activities relevant to human health [75]. Based on structural variations in the C ring, flavonoids are categorized into several subclasses, including flavones, flavonols, flavanones, flavan-3-ols (catechins), anthocyanidins, and chalcones (Fig. 1) [76-80].

Although many flavonoids share antioxidant and anti-inflammatory capacities, each compound can also engage specific molecular targets [81]. These activities have been linked to potential benefits in several age-related conditions, particularly those driven by oxidative stress and chronic inflammation (Table 2*)* [82].

### 2.1.1 Flavonoids and age-related diseases

Because of their antioxidant, anti-inflammatory, and neuroprotective qualities, flavonoids have attracted a lot of interest for their potential to slow the onset and progression of certain age-related diseases *(Fig. 2)* [78, 79, 83-90].

**Figure 2.**
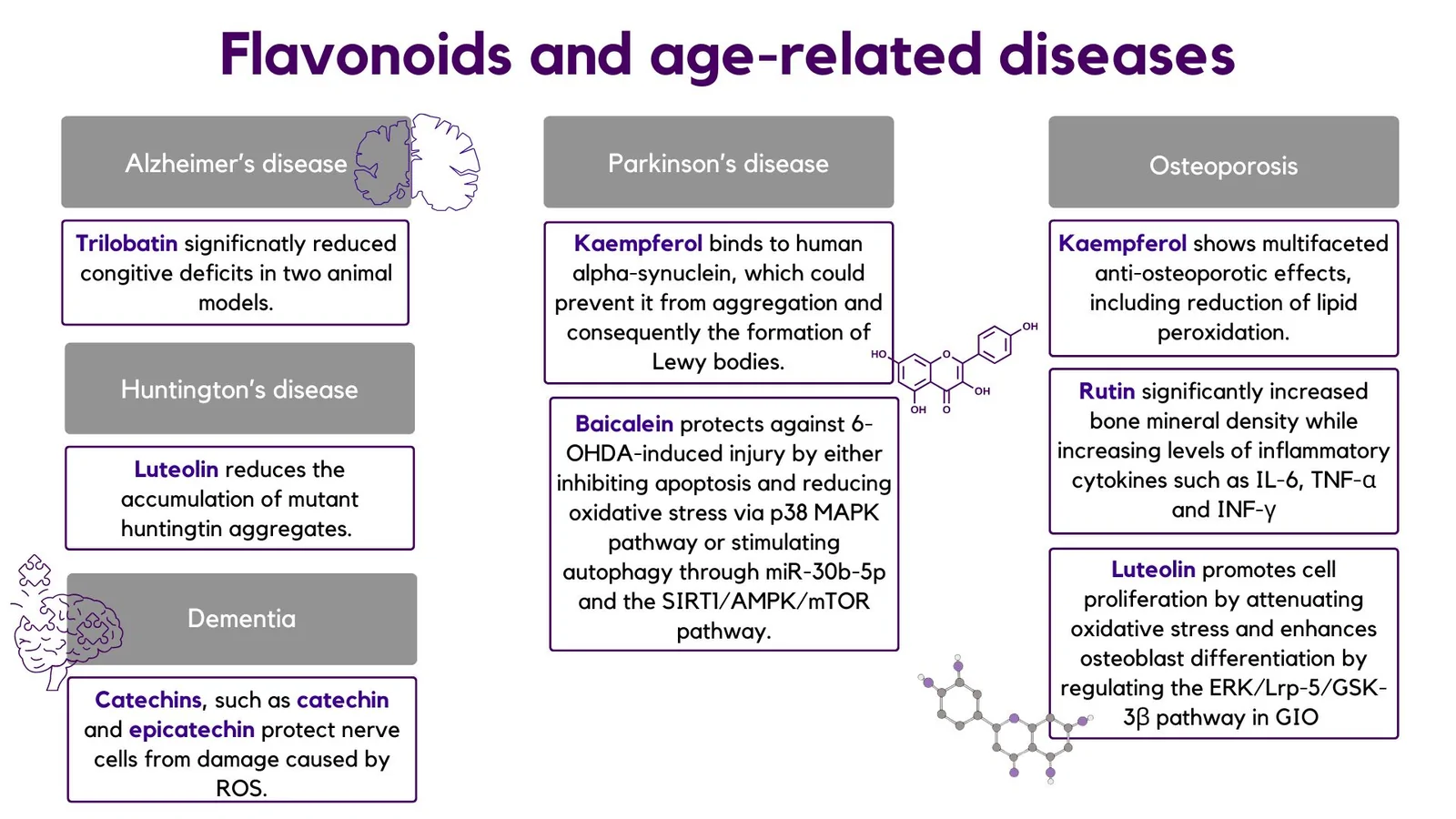
Examples of flavonoids that exhibit potential to mitigate the progression of specific age-related diseases and the leading mechanisms of their activity [78, 79, 83-90].

### 2.1.1.1 Trilobatin in the treatment of Alzheimer's disease

Aging is one of the key risk factors for the development of Alzheimer's disease (AD) [91]. During the course of this disease, a pathological accumulation of intracellular neurofilament tangles and the deposition of β-amyloid peptide outside cells occur [92]. Such effects are significantly associated with oxidative stress, as previously mentioned, flavonoids can reduce oxidative stress, thereby protecting cells from free radical damage [93],[81]. Trilobatin is also worth noting in this aspect. It constitutes a flavonoid isolated from *Lithocarpus polystachyus* leaves, which has been investigated in Alzheimer’s models [94, 95]. Jian-mei Gao et al. conducted a study to discover the molecular mechanisms behind the beneficial effects of TLBs in experimental models of AD, both *in vivo* and *in vitro*. The results showed that the use of TLB at different doses significantly reduced cognitive deficits in two animal models of AD, as assessed by the open-field test, the novel object recognition test, the Y-maze test, and the Morris water maze test, among others. Findings suggest that it counters Aβ25-35 toxicity by enhancing the SIRT3/SOD2 axis, which restores redox balance and reduces neuroinflammatory responses. Thus, redox homeostasis was restored, and neuroinflammatory processes were attenuated [96].

### 2.1.1.2 Dementia

Tea-derived catechins, particularly catechin (CA) and epicatechin (EC), have been shown to mitigate oxidative injury in neuronal models, implying a protective role against dementia-related processes [97]. In mouse experiments, EC ameliorated glucose oxidase-induced memory impairment, and both CA and EC alleviated memory deficits resulting from brain ischemia [97]. These findings suggest the potential use of tea catechins in protecting against senile neurodegenerative disorders such as dementia. A. Jennings et. al. performed a study involving 121,986 people, which showed that increased intake of flavonoids, present in berries, tea, and red wine, could reduce the risk of dementia by 28%. This effect was particularly pronounced in people with high genetic risk and depressive symptoms [98]. Another study on 1,367 people over 65 years of age observed that higher flavonoid intake was associated with a lower risk of dementia (RR=0.49 after adjustment for demographic and dietary factors, p=0.04) [99].

### 2.1.1.3 Parkinson's disease

Parkinson's disease constitutes a complex, progressive ND, first described in 1817 by James Parkinson [100]. It is characterized by the loss of dopaminergic neurons in the compact substantia nigra pars compacta and the presence of Lewy bodies containing aggregates of alpha-synuclein. It affects more than 1 percent of people over 60 years of age, and the incidence rises to 5 percent in people over 85 years of age [101]. With age, substantia nigra neurons become more susceptible to cell death due to the accumulation of mitochondrial DNA defects, oxidative damage, and neuromelanin accumulation. These factors increase the vulnerability of neurons, and additional stress, such as toxic alpha-synuclein or mitochondrial dysfunction, leads to neuronal death. The cumulative effect of these processes contributes to the degeneration of substantia nigra neurons in Parkinson's disease [102].

Baicalein, the main flavonoid of the roots of *Scutellaria baicalensis* Georgi, exhibits antimicrobial, antiviral, anti-inflammatory, antioxidant, and anticancer activities [103]. Min Chen and co-authors performed a study on rats in which a model of Parkinson's disease was obtained by injecting 6-OHDA into the left striatum, followed by baicalein treatment [104]. The study demonstrated that baicalein protected PD rats by stimulating mitochondrial autophagy through the miR-30b-5p and SIRT1/AMPK/mTOR pathways.

Another flavonoid that proves significant in terms of PD management is kaempferol (3,5,7-trihydroxy-2-(4-hydroxyphenyl)-4H-chromen-4-one), which was discovered in Camellia sinensis (tea tree) [105]. This chemical compound exhibits anti-cancer, anti-inflammatory, anti-adipogenic, hepatoprotective, antioxidant, antihistamine, and antimicrobial properties [106]. Rahul and co-authors performed a study to investigate the effect of kaempferol on a transgenic model of Parkinson's disease in fruit flies. The results showed that kaempferol delayed the loss of climbing ability and improved the activity of PD flies in a dose-dependent manner, compared to PD flies without kaempferol. A dose-dependent reduction in oxidative stress markers was also observed. Molecular docking showed that kaempferol binds to human alpha-synuclein, which could prevent its aggregation and consequently the formation of Lewy bodies [107].

### 2.1.1.4 Huntington's disease

Huntington's disease (HD) is a progressive ND inherited in an autosomal dominant manner that eventually leads to death. It is caused by an expansion of CAG repeats in the huntingtin gene, which leads to the synthesis of the mutant HTT protein [108]. Symptoms of HD usually appear in people between 30 and 50 years of age and increase with age [109]. The positive effects of luteolin in HD are noteworthy. Luteolin (3′,4′,5,7-tetrahydroxyflavone; C_1__s_H_1__o_O_6_) is a flavone belonging to the flavonoid group, characterized by its yellow color [110]. It is found in vegetables of the celery family, such as carrots and parsley, as well as broccoli, spinach, cabbage, and green chili peppers [111]. A. Ramadan et al. demonstrated the neuroprotective effects of luteolin in HTT mutant neuroblastoma cells in their study. They showed that luteolin protects cultured cells from the cytotoxic effects of mutant huntingtin, increasing their viability and reducing apoptosis. Additionally, it reduces the accumulation of both soluble and insoluble aggregates of this protein in neuroblastoma cells [112]. Another study found that HD model mice treated with luteolin exhibited improved motor coordination and balance, as well as a significant reduction in motor impairment. In addition, luteolin reduced the levels of neurofilament light chain (a biomarker of neurological disorders [113]) in the serum of HD mice. Importantly, luteolin-treated mice showed a significant reduction in the accumulation of huntingtin aggregates in the cerebral cortex, hippocampus, and striatum [114].

### 2.1.1.5 Osteoporosis

Osteoporosis is one of the most prevalent age-related conditions, affecting about 30% of women and 8% of men over the age of 50. Its burden is expected to grow, with fracture incidence in the six largest EU countries projected to reach 3.3 million cases by 2030 [115]. Low peak bone mass is associated with an increased risk of osteoporotic fractures, although its role is not yet fully understood [116]. Peak bone mass is an important determinant of bone density in old age [116, 117]. In addition, there is a natural loss of bone mass with age, which is also a risk factor for osteoporosis [117, 118].

Kaempferol, previously described as an adjunct in the treatment of PD [104-107], has also shown anti-osteoporotic effects. The mechanism includes: (a) inhibition of adipogenesis in favor of osteoblastogenesis and chondrogenesis, (b) activation of the estrogen receptor pathway, (c) upregulation of Runx-2 expression in BMP-2 signaling, (d) inhibition of NF-κB and the inflammatory response, (e) reduction of ROS and lipid peroxidation, (f) regulation of autophagy in osteoblasts and osteoclasts, (g) inhibition of osteoblast apoptosis, and (h) modulation of proteins involved in osteoblast mineralization [119]. Z. Jing and colleagues noted that in glucocorticosteroid-induced osteoporosis, luteolin promotes cell proliferation by attenuating oxidative stress and enhances osteoblast differentiation by regulating the ERK/Lrp-5/GSK-3β pathway in GIO [120]. Naringin, a natural flavanone glycoside found in citrus, grapes, cherries, tomatoes, and oregano [121, 122], exhibits anti-osteoporotic effects by inhibiting osteoclastogenesis. The mechanism involves modulation of RANK/RANKL interaction and induction of osteoclast apoptosis [40]. A study by Pang et al. showed that naringin treatment in OVX mice improved femoral, tibial, and lumbar bone quality, increased bone strength, and reduced urinary calcium excretion [123].

### 2.1.2 Quercetin and SIRT1 in the context of aging

Quercetin (2-(3,4-dihydroxyphenyl)-3,5,7-trihydroxy-4H-1-benzopyran-4-one) is a natural flavonoid found in both fruits (e.g., grapes, peaches) and vegetables (e.g., onions, garlic). Quercetin is highly soluble in lipids and alcohol but completely insoluble in water. It is an aglycone, but the attachment of a glycosyl group at position 3 (replacing the hydroxyl group) leads to the formation of quercetin glycoside, which is water-soluble. The bioavailability of quercetin in humans is low, with a half-life of 1-2 hours after ingestion [124]. This flavonoid is absorbed by passive diffusion or with the help of an organic anion-transporting polypeptide from the small intestine and then metabolized in the liver, intestines, and kidneys, resulting in its rapid degradation [125]. For this reason, pharmacological effects are mainly seen in *in vitro* rather than *in vivo* studies. The aging process is associated with inherent structural and functional changes within neuronal circuits, leading to a decrease in the number of dopamine receptors, activation of microglia and astrocytes, and an excessive pro-inflammatory response, resulting in chronic inflammation [126]. Sirtuin 1 (SIRT1) attenuates the degeneration of dopaminergic neurons and regulates the expression of proinflammatory cytokines [127]. Several studies have shown that quercetin protects against NDs by enhancing SIRT1 deacetylase activity [128, 129].

### 2.1.2.1 Regulation of oxidative stress via SIRT1

J. Papaconstantinou revealed a significant correlation between oxidative stress and the aging process, as well as age-related diseases such as cardiomyopathy [130]. Quercetin increases antioxidant enzymes and anti-inflammatory cytokines and improves mitochondrial function by regulating SIRT1 activity. It acts by the following signaling pathways: SIRT1/AMPK/NF-κB, SIRT1/Keap1/Nrf2/HO-1, or SIRT1/PI3K/Akt [92, 94]. In addition, quercetin has been observed to inhibit oxLDL-induced endothelial oxidative damage through activation of SIRT1 and modulation of the AMPK/NADPH oxidase/AKT/ endothelial NO synthase pathway [131]. In an experimental model of streptozotocin (STZ)-induced diabetes, quercetin caused a significant increase in SIRT1, SOD, and CAT activity in kidney tissues, which contributed to a reduction in oxidative damage in liver and kidney tissues and a decrease in NF-κB levels [132].

### 2.1.2.2 Regulation of the inflammatory response and effects on mitochondria by SIRT1

One anti-aging strategy is to mitigate the inflammatory response. It is worth noting that, of all organelles, mitochondria and lysosomes are the most susceptible to aging-related changes. Quercetin, by modulating SIRT1 activation via the AMPK/SIRT1/Nrf2/TNFα pathway, contributes to the anti-inflammatory effect [133]. Mitochondrial dysfunction influences the development of aging-related diseases [134, 135], as mitochondria play a key role in the production of ROS and cell death processes [136]. Quercetin inhibits oxLDL through activation of SIRT1 and modulation of the AMPK/NADPH/AKT oxidase pathway [131] and reduces ROS production in mitochondria through the SIRT1/Nrf2/HO-1 pathway [137].

**Table 2:** Summary of flavonoids and their biological effects.

Flavonoid	Mechanism of action	Effects on the organism
**Trilobatin**	Inhibition of HMGB1 acetylation, activation of the SIRT3/SOD2 pathway	Reduction of cognitive deficits in AD, restoration of redox homeostasis
**Catechins**	Protection from oxidative stress	Improved memory, reduced risk of dementia
**Baicalein**	Inhibition of apoptosis, reduction of oxidative stress via p38 MAPK	Neuronal protection in PD
**Kaempferol**	Binding to alpha-synuclein, inhibiting its aggregation Activation of the estrogen receptor pathway, reduction of oxidative stress	Delaying the symptoms of PD, Supporting osteoporosis treatment
**Luteolin**	Protection of nerve cells, reduction of huntingtin aggregates	Improving motor function in HD
**Rutin**	Increase bone mineral density	Improving bone structure in osteoporosis
**Naringin**	Modulation of RANK/RANKL interaction, induction of osteoclast apoptosis	Inhibition of osteoclastogenesis, improvement of bone quality
**Quercetin**	Increases SIRT1 activity through the SIRT1/AMPK/NF-κB, SIRT1/Keap1/Nrf2/HO-1, and SIRT1/PI3K/Akt pathways; increases levels of antioxidant enzymes and inhibits oxidative damage, including in the endothelium	Protection against aging and NDs - reducing inflammatory reactions, improving mitochondrial function, and lowering the production of ROS

Through mechanisms like SIRT1 or SIRT3 activation and NF-κB suppression, which affect telomerase activity and shield telomeric DNA from oxidative damage, a number of flavonoids indirectly modify telomere biology [92, 94, 134, 140]. Docking studies for kaempferol suggest possible interactions with telomeric proteins, despite the lack of a thorough mapping of direct binding sites on telomerase [109]. In neurodegeneration models, luteolin and trilobatin provide neuronal protection, and in bone, kaempferol and naringin stimulate osteo-blastogenesis, demonstrating tissue-specific effects [109, 121, 126]. It is difficult to predict the effects of flavonoids and optimize their therapeutic use due to their complex and incompletely understood mechanisms of action. The majority of the evidence is derived from animal models and in vitro studies, with carefully planned clinical trials attesting to their effectiveness. Evaluation is made more difficult by the lack of standardization with regard to source, dose, and formulation, and their effects differ significantly between individual compounds and experimental models. Furthermore, many flavonoids have low bioavailability. For example, quercetin's clinical utility is limited due to its rapid metabolism (t½ ≈ 1-2 h), and near-insoluble nature in water [138, 139]. The safety of long-term use and possible drug interactions must also be carefully considered. These effects are supported by a large number of mechanistic and preclinical studies, but there is a dearth of human data. There have only been a few randomized controlled trials, and they frequently have variable formulations, heterogeneous endpoints, and small sample sizes. This restricts the capacity to offer firm therapeutic advice. The practical use of flavonoids hinges on a better understanding of their mechanisms, methods to increase their bioavailability, and strong clinical evidence proving their safety and benefits, despite the fact that they show promising antioxidant and neuroprotective qualities.

### 2.2 Carotenoids

Carotenoids are a large class of lipophilic pigments present in fruits, vegetables, algae, and photosynthetic microorganisms [140, 141]. In plants, they are responsible for yellow, red, orange, and pink coloration [142]. In addition to the shared antioxidant and mitochondrial-protective mechanisms outlined earlier, carotenoids have neuroprotective effects through anti-apoptotic pathways and may promote neural plasticity [143]. Structurally, they are divided into carotenes (e.g., α-carotene, β-carotene) and xanthophylls (e.g., lutein, zeaxanthin, fucoxanthin, violaxanthin, antheraxanthin, neoxanthin) [144]. Carotenoids perform a wide range of functions in the human body, including enhancement of cognitive function, support for cardiovascular health, and anticancer properties [140, 145, 146]. Those compounds are obtained through diet and accumulate in the skin, where they effectively protect against UV radiation and related damage. Another key function of carotenoids is the enhancement of the transcription of antioxidants and detoxifying enzymes. Additionally, they influence telomere length, as studies demonstrate a correlation between higher dietary intake of beta-carotene and longer telomeres [147]. Nevertheless, according to certain research, taking supplements of lutein, lycopene, or beta-carotene does not alter the activity of antioxidant enzymes or increase LDL resistance to oxidation [148]. This emphasizes the necessity of more study in this field. There are restrictions on the use of carotenoids. Because of their synergistic interactions with other compounds, they may be less effective when used alone. However, consuming several carotenoids at once may cause competition for absorption, which would reduce bioavailability. Additionally, carotenoids are relatively unstable and can easily be converted into other compounds, further complicating their efficacy and consistent biological activity [149]. Other studies have demonstrated the role of carotenoids in improving cognitive function. Higher carotenoid intake has been generally associated with a lower risk of Alzheimer's disease [150]. However, there are no pharmacological therapies involving carotenoids, only dietary recommendations, due to the lack of sufficient evidence establishing a strong correlation between carotenoid intake and cognitive enhancement [151].

### 2.2.1 Telomere length

Telomeres are repetitive DNA-protein complexes at chromosome ends that safeguard genomic stability by preventing fusion and degradation. Their length naturally declines with cell division and is further shortened by oxidative and replicative stress influenced by genetic and environmental factors. Telomerase activity can counteract this process by extending telomeres, thereby supporting cellular longevity [152-159].

It has been demonstrated that daily intake of α-carotene or β-carotene positively influences the telomere length of buccal cells [160]. Scientific studies also indicate that higher blood levels of alpha-carotene, beta-carotene (trans + cis), and beta-cryptoxanthin are associated with telomere lengths up to 5-8% longer [161]. Interestingly, in individuals who are overweight or obese, an increase in beta-carotene levels is also correlated with an increase in telomere length [162]. We can therefore infer that higher carotenoid intake is associated with longer telomere length [163].

Telomere shortening is a hallmark of cellular aging and a common feature of age-related diseases [158]. It can be stated that shorter telomere length triggers a mechanism that drives replicative aging by limiting the mitotic potential of cells [164]. Several hundred nucleotide repeats at the end of each chromosome are required to prevent the activation of DNA repair pathways [164]. Therefore, we should consume foods that ensure a high level of carotenoids in order to help prevent age-related diseases. A high dietary intake of zeaxanthin and lutein is likely to offer protection against age-related conditions, such as age-related macular degeneration (AMD) [165, 166].

### 2.2.2 Carcinogenesis

Altered telomere length disrupts chromosomal integrity and has been implicated in carcinogenesis [167]. Both very short and abnormally long telomeres can promote tumorigenesis, depending on context [168]. In fact, telomere abnormalities have been reported in the vast majority of early epithelial cancers, with shortening as the most common alteration [169]. Diet is a major modifiable lifestyle factor that may contribute to the regulation of cancer risk by as much as 40%. Data suggest that several carotenoids may prove beneficial in reducing carcinogenesis [170]. There is evidence that various carotenoids, such as β-carotene, α-carotene, lycopene, lutein, zeaxanthin, β-cryptoxanthin, fucoxanthin, canthaxanthin, and astaxanthin, exhibit anticancer properties in certain tissues. There are indications that carotenoids, in combination with other antioxidants, could potentially be used as a preventive measure against cancer [171]. However, this topic requires further investigation, as there are reports of interactions between β-carotene and substances such as cigarette smoke and alcohol, which may lead to effects that differ from the intended therapeutic outcomes [172-174]. It has been demonstrated that carotenoids exhibit pro-oxidant activity in cancer cells and antioxidant activity in healthy cells, which presents significant potential for their application, considering the critical role of oxidative stress in carcinogenesis [145]. Carotenoids are undoubtedly promising compounds in the context of cancer prevention; however, their application still requires further research.

Carotenoids such as β-carotene and lutein have been associated with longer telomere length in buccal cells and peripheral leukocytes [163-166]. The mechanisms are mostly ascribed to the modulation of redox-sensitive transcription factors that control the expression of telomerase and the antioxidant protection of telomeric DNA. Tissue-level evidence supports roles in retinal protection, cardiovascular function, and cognitive preservation, despite the lack of binding affinity data for telomerase [150, 168-169]. Carotenoids have been shown to have health benefits, but a number of issues limit their practical use. Because of their lipophilic nature, reliance on dietary fat for absorption, and inter-individual metabolic variability, poor bioavailability is still a major concern [140]. What’s important is that clinical results vary, with some research showing no discernible impact on disease biomarkers or oxidative stress [148]. Furthermore, high-dose supplementation, especially in synthetic forms, has been linked to possible negative effects, such as a higher risk of some types of cancer in smokers [173]. Differences in carotenoid type, dosage, source, and study design make comparisons even more difficult and prevent the development of standardized recommendations [165].

### 2.3 Proteins and peptides

Proteins and peptides affect cell cycle regulation and particular age-related signaling pathways in addition to the well-known anti-oxidative and mitochondrial-supportive mechanisms. Recent studies emphasize the roles of mitochondrial-derived peptides and regulatory proteins such as p53 and p21 in maintaining homeostasis and delaying age-associated decline (Fig. 3) [175-184].

**Figure 3.**
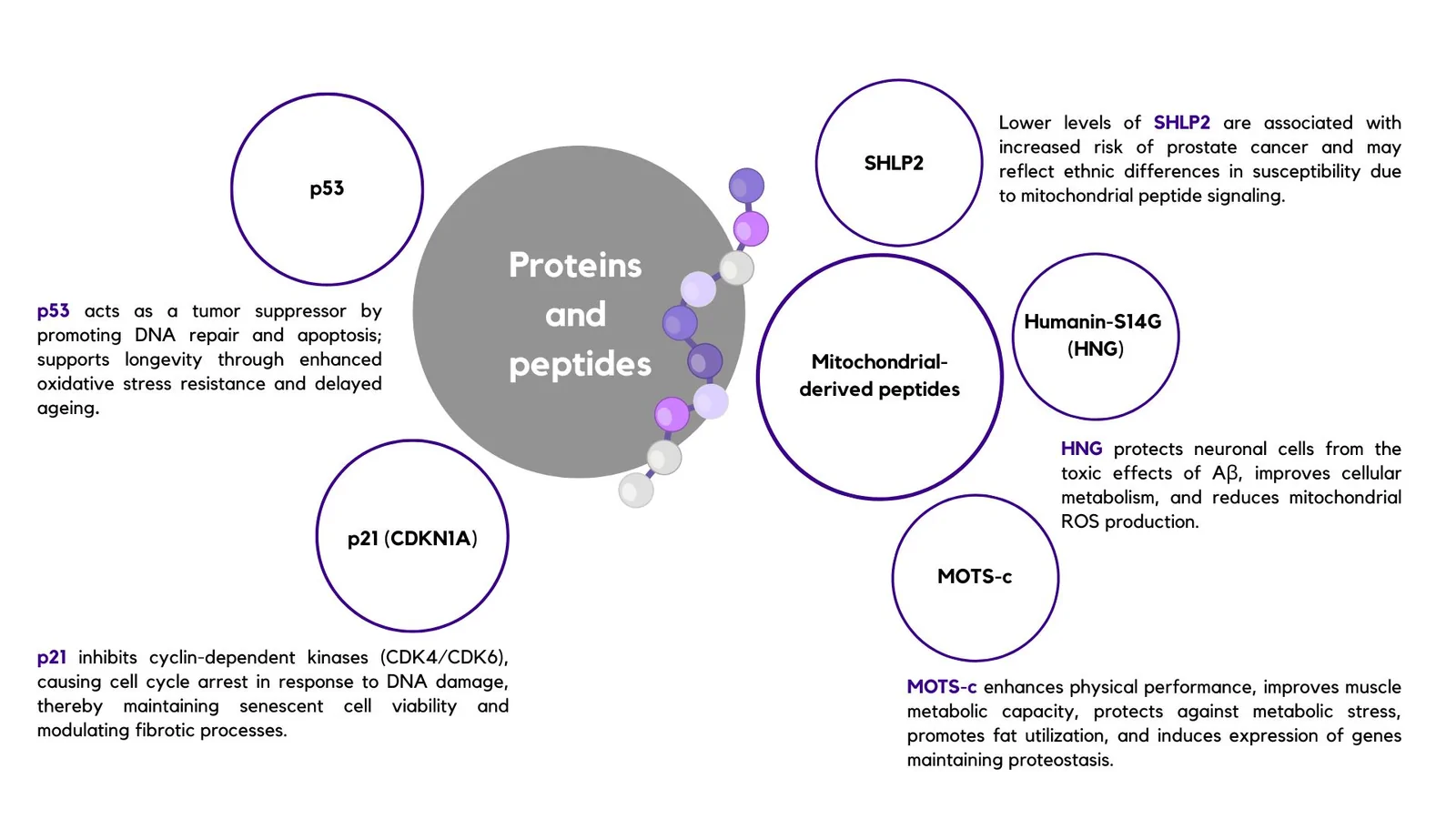
**Examples of proteins and peptides that take part in the regulation of cellular processes connected with aging and the leading mechanisms of their action**. Representative nuclear proteins and mitochondrial-derived peptides involved in longevity-related pathways. Tumor-suppressors p53 and p21 govern DNA-damage repair, apoptosis, and cell-cycle arrest, while the mitochondrial peptides SHLP2, Humanin-S14G, and MOTS-c fine-tune metabolic homeostasis, neuroprotection, and oxidative-stress resistance, together shaping cellular aging paths [175-184].

### 2.3.1 Mitochondrial-derived peptides

Recently discovered mitochondrial-derived peptides (MDPs) act as mediators of mitochondrial feedback signaling [185]. As the body ages, levels of these peptides decline, resulting in impaired physiological function [185, 186]. At the same time, reduced MDPs levels are associated with the development of diseases characteristic of aging [187, 188].

An example of an MDPs is humanin, identified in 2001 from a cDNA library of surviving neurons in an Alzheimer’s patient [189] Humanin protects against oxidative stress, nutrient deprivation, and hypoxia in vitro, and has shown cardioprotective and neuroprotective effects *in vivo* [190]. Studies have shown that humanin-S14G (HNG) has a neuroprotective effect, protecting neuronal cells from the toxic effects of Aβ, improving their metabolism, and reducing the production of ROS in mitochondria. In experiments on aged mice, HNG enhanced cognitive function, as evidenced by, among other things, better performance in tests of balance, memory, and spontaneous alternation, while reducing microglia activation and lowering inflammatory markers. Additionally, an interesting finding was that in humans, African-Americans were found to have lower levels of humanin than whites, and the SNP variant rs2854128 was associated with reduced levels of humanin and accelerated cognitive aging, particularly in the African-American population [191]. Interestingly, Xiao J et al. showed that a second mitochondrial peptide, SHLP2, is also lower in African-Americans compared to Caucasian Americans, and reduced levels predict prostate cancer risk [192]. Another one of MDPs, known as MOTS-c, was detected in 2015 [186]. In people aged 70-81 years, MOTS-c concentrations are about 21% lower than in young adults in the 18-30 age group [193]. Other studies also show a correlation between reduced MOTS-c and the aging process [194, 195]. A study by Joseph C Reynolds and co-authors showed that MOTS-c responds to exercise, as evidenced by increased levels in the muscle and blood of healthy young men after exercise. In experiments on mice, both young and old, administration of MOTS-c improved physical performance, increased muscle metabolic capacity, and had beneficial effects on body composition, including reducing fat gain and increasing muscle mass. Additionally, *in vitro* studies on muscle cells showed that MOTS-c protects cells from metabolic stress, improves fat utilization, and induces the expression of genes related to the maintenance of protein homeostasis [196]. Overall, the results suggest that MOTS-c plays an important role in adaptation to exercise-induced stress, which may contribute to improved physical ability and metabolic health, and the strong correlation between pathological outcomes at different ages and MOTS-c levels suggests that higher levels of MOTS-c are beneficial for delaying aging [197].

### 2.3.2 p53 and p21

Regulatory proteins such as p53 and p21 play central roles in aging and longevity [198]. Studies in animal models provide convincing evidence that the functional p53 pathway promotes longer life [199]. In animal studies of young adults, low and comparable levels of p53 were observed between groups, whereas a significant increase was observed in older mice, especially in the wild-type group [200]. In addition, a decrease in p53 activity has been reported in aging mice, which is associated with an increased risk of cancer and a shortened lifespan [201]. In contrast, mice carrying extra copies of the Arf and p53 genes show better protection against cancer, less oxidative damage, and delayed aging [202], while mice with the p53S18A mutation, associated with a defect in the p53 pathway, are characterized by accelerated aging and earlier cellular senescence [203]. These findings highlight the critical importance of proper p53 pathway function for maintaining longevity. The p21 protein (CDKN1A) acts mainly by binding to and inhibiting the activity of cyclin-dependent kinases, particularly CDK4 and CDK6, which prevents the phosphorylation of retinoblastoma proteins and inhibits the expression of proliferation-related genes. This results in sustained cell cycle arrest, which is a defense mechanism against tumor development and promotes tissue repair [204]. Surprisingly, elevated levels of p21, which induce cell cycle arrest in senescent cells, simultaneously maintain their viability, especially when senescence is caused by DNA damage. The persistent DNA damage response (DDR) is a key molecular mechanism mediating aging and age-related diseases, leading to increased p21 expression. Importantly, downregulation of p21 in senescent cells exacerbates DDR, a result of these cells attempting to re-enter the cell cycle. This enhancement is accompanied by increased activation of telangiectasia mutated ataxia and nuclear factor kinase NF-κB, whose activation induces TNF-α secretion and JNK activation, leading to senescent cell death in a caspase- and JNK-dependent manner [205]. Mice lacking p21 exhibit less fibrosis, which may be due to both increased apoptosis of senescent cells and less senescent cell formation resulting from reduced TGF-β signaling activity and collagen production. Consequently, p21 inhibition not only promotes the elimination of senescent cells but may also reduce fibrotic processes and improve tissue regeneration by increasing the proliferative capacity of stem and progenitor cells [205]. Therefore, controlled modulation of p21 activity may represent a promising therapeutic strategy for the treatment of both fibrotic pathologies and other aging-related conditions, while contributing to improved tissue function and prolonged healthy lifespan.

By lowering oxidative stress and modifying SIRT pathways, mitochondrial-derived peptides like humanin and MOTS-c may have an indirect effect on telomere stability [193, 199]. These peptides have tissue-specific advantages such as enhanced muscle function, cardiovascular protection, and neuroprotection in Alzheimer's models, despite the fact that their telomerase binding sites are unknown [193, 194, 199]. There are a number of significant obstacles to the use of proteins and peptides in anti-aging treatments. Peptides and proteins frequently have brief biological half-lives, are vulnerable to proteolytic breakdown, and pose challenges for targeted delivery, especially to the central nervous system or mitochondria. They might also have unexpected systemic effects and immunogenicity risks. Standardized and optimized formulations, rigorous clinical trials, and a deeper understanding of the mechanisms of action of promising preclinical findings are all necessary to translate them into safe, effective, and well-tolerated long-term therapies in humans.

### 2.4 Polysaccharides

Polysaccharides from natural sources demonstrate a wide range of biological activities, including antioxidant, anti-inflammatory, and immunomodulatory effects (Fig. 4) [206, 212-218]. Some of them, such as heparin, are already used in clinical practice [207], while others sow promising results in preclinical studies [208-211].

### 2.4.1 Angelica sinensis

*Angelica sinensis* (ASP), a major bioactive component of dong quai, has been reported to exert antioxidant, immunomodulatory, and neuroprotective effects [219]. In a D-galactose-induced aging model, ASP improved hematopoietic function by modulating oxidative stress, DNA damage, and related signaling pathways. This may have potential implications for anti-aging therapies and improving bone marrow function [220]. ASP has shown significant effects on brain aging, particularly in the context of D-galactose-induced changes. Its administration improves learning ability and spatial memory in rats, demonstrating its beneficial effects on cognitive function. At the same time, it leads to a reduction in the number of aging cells in brain tissue, as confirmed by the results of SA-β-Gal staining. At the molecular level, ASP affects the length of telomeres and telomerase activity, suggesting that it may protect cells against telomere shortening, one of the primary mechanisms of aging. By combining these mechanisms, ASP shows potential in slowing brain aging and protecting neurons from neurodegenerative processes. It is a promising candidate for further research into anti-aging strategies in the nervous system [210]. In a study in mice in which aging was induced with D-galactose, ASP effectively counteracted the degeneration of testicular tissue by limiting structural damage and reducing the decline of Leydig and spermatogonial cells. One of ASP's key mechanisms of action is its potent antioxidant effect - administration of this substance reduced malondialdehyde (MDA) levels, a marker of oxidative stress, while increasing the activity of antioxidant enzymes (SOD, TAOC). The protective effect of ASP was also confirmed at the hormonal level, where the substance inhibited the decline in testosterone levels typically associated with the aging process. The results of this study suggest that ASP may be an effective anti-aging agent, especially for the protection of the reproductive system and endocrine function [211]. In addition, it regulates the expression of aging-related genes, including p19, p21, and p53, whose excessive activity accelerates degenerative processes in cells [210, 211].

**Figure 4.**
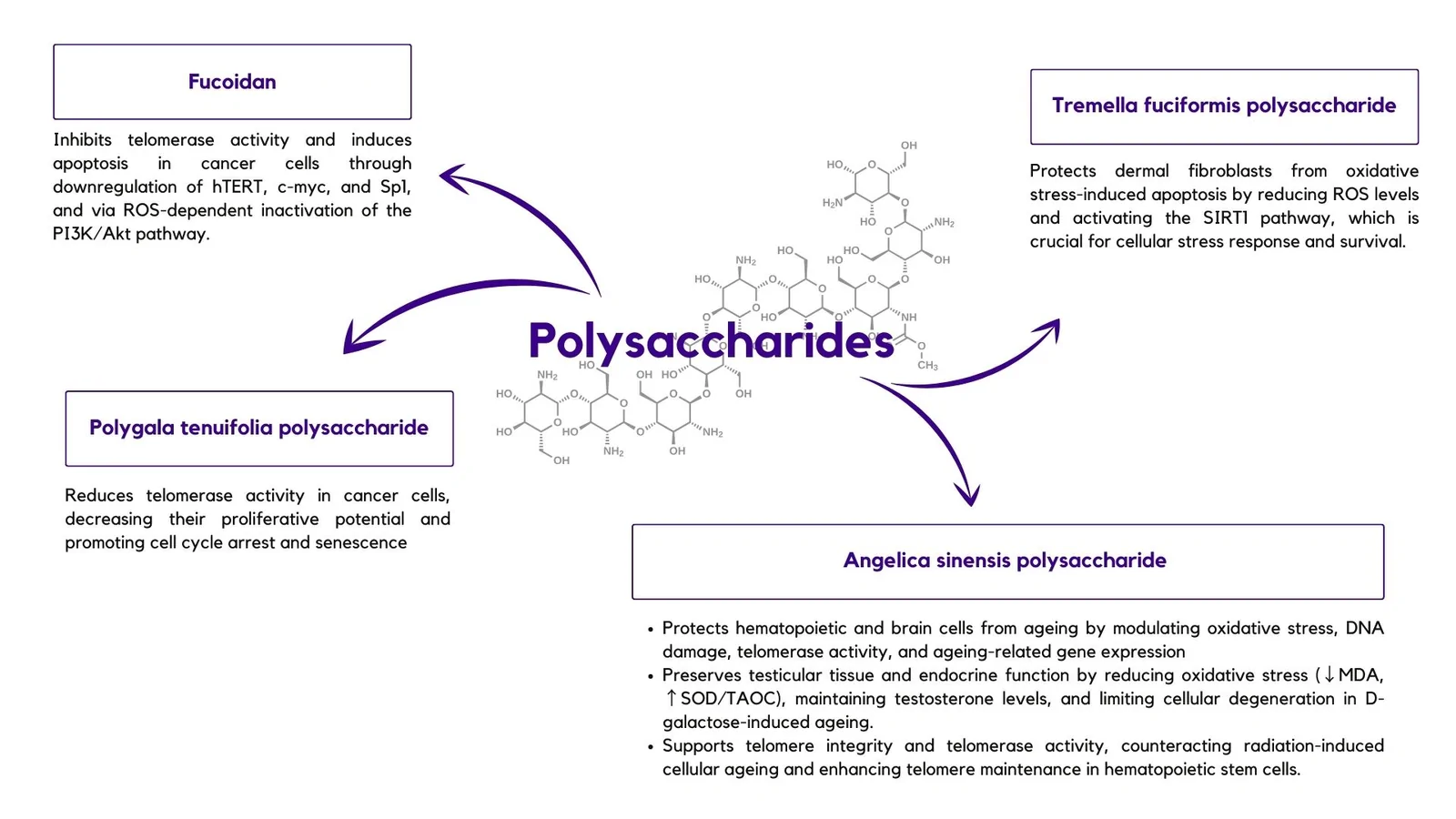
**Bioactive polysaccharides that modulate key hallmarks of aging**. Marine-derived fucoidan and Polygala tenuifolia polysaccharide blunt telomerase (hTERT) signaling, down-regulate oncogenic drivers (c-Myc, Sp1) and PI3K/Akt activity, thereby enforcing cell-cycle arrest and senescence in rapidly dividing cells. The fungal β-glucan from Tremella fuciformis shields dermal fibroblasts by scavenging ROS and activating the SIRT1 stress-response pathway. Angelica sinensis polysaccharide protects haematopoietic, neural, and endocrine tissues by limiting oxidative DNA damage, maintaining testosterone levels, and preserving telomere length and telomerase competence following radiation or D-galactose challenge. Together, these structurally diverse glycans illustrate how redox control, telomere maintenance, and cell-cycle regulation converge to confer polysaccharide-driven longevity benefits [212-218].

### 2.4.2 Tremella fuciformis polysaccharide

Polysaccharides extracted from *Tremella fuciformis* (TFPS) display antioxidant, anti-inflammatory, and cytoprotective activities in experimental systems [221, 222] and thus have found their clinical application in China for cancer treatment and anti-aging therapies [223]. The results of this study indicate that TFPS exhibits protective properties against oxidative stress-induced damage and apoptosis in human dermal fibroblasts exposed to hydrogen peroxide. TFPS reduces the production of ROS and inhibits apoptosis processes in fibroblasts, leading to improved survival. Activation of the SIRT1 pathway, which is crucial for protecting cells against oxidative stress, plays a significant role in this process. The use of the SIRT1 inhibitor, niacinamide, completely attenuated the protective effect of TFPS, confirming that SIRT1 activation is essential for a positive impact [224]. This suggests that TFPS may have potential applications in delaying aging processes in the context of skin aging.

### 2.4.3 Polysaccharides in the modulation of telomerase activity

Fucoidan, a polysaccharide from brown algae, has been shown to suppress telomerase by downregulating hTERT and transcription factors such as c-myc and Sp1 [225]. This effect is linked to inhibition of the PI3K/Akt pathway and increased ROS production, suggesting a dual role in promoting apoptosis and limiting uncontrolled proliferation. The results indicated that fucoidan induces apoptosis and inhibits telomerase in a ROS-dependent manner through inactivation of the PI3K/Akt pathway [226]. In contrast, another polysaccharide, PTP isolated from the roots of Polygala tenuifolia, leads to a decrease in telomerase activity in cancer cells, which may contribute to a reduction in their proliferative potential and ability to grow uncontrollably [227]. X-ray was also found to significantly increase the number of cells in the G1 phase of the cell cycle, increase aging-associated β-galactosidase (SA-beta-Gal) levels and p53 protein expression, while shortening telomere length and decreasing telomerase activity. ASP administration during radiation exposure resulted in the inhibition of these changes, reducing the number of cells in the G1 phase, decreasing p53 expression, and the number of SA-beta-Gal-positive cells, as well as lengthening telomeres and increasing telomerase activity. This suggests that ASP may have a protective effect on HSCs by delaying the aging process, which may be due to its effects on telomere stability and the regulation of molecular mechanisms related to the cell cycle and apoptosis. Thus, ASP may represent a promising means to protect stem cells and potentially slow down the body's aging process [228].

Fucoidan is one of the polysaccharides that directly suppresses hTERT expression and inhibits telomerase by suppressing the PI3K/Akt pathway [229]. On the other hand, polysaccharides from Angelica sinensis have been demonstrated to increase telomerase activity and maintain telomere length in hematopoietic stem cells [213, 231]. Neuroprotection, testicular preservation, and bone marrow function support are examples of tissue-specific effects [213-214]. The lack of binding site mapping at this time indicates a knowledge gap. The majority of evidence supporting polysaccharides' broad pharmacological potential comes from in vitro tests or animal models, which restricts the direct application of these findings in clinical settings, despite multiple studies confirming this. Their intricate molecular makeup and source-dependent composition make standardization extremely difficult, which in turn makes it difficult to compare study findings. Only a small amount of the underlying molecular mechanisms are known, especially those pertaining to telomerase modulation and effects on cellular senescence. The lack of extensive clinical trials confirming these compounds' long-term safety and effectiveness is another major disadvantage.

### 2.5 Essential Oils components

The International Organization for Standardization (ISO) defines an essential oil as a product manufactured by either water or vapor distillation, by mechanical processing of citrus rinds, or by dry distillation, which are commonly used in cosmetic products [229]. Specific constituents such as linalool, β-caryophyllene, and limonene have been shown in experimental models to exert anticancer, antimicrobial, antioxidant, anti-inflammatory, or neuroprotective effects, though the strength of evidence varies depending on compound and study design [230]. Due to anti-inflammatory properties relevant to various inflammatory conditions such as respiratory inflammation, atopic dermatitis, and arthritis, specific compounds have shown potential in alleviating symptoms and targeting disease-specific pathways, suggesting their therapeutic potential *(Fig. 5)* [231-239].

Diterpenes and other components of essential oils work through compound-specific mechanisms in addition to the general antioxidant effects that have already been discussed. Diterpenes raise the amounts of endogenous antioxidant enzymes and prevent the synthesis of pro-inflammatory mediators. Diterpenes are used in skin care products to postpone aging [240] and have been shown to have neuroprotective properties [241]. Some of the triterpenoids- a type of terpenes isolated from the dried fruiting bodies of *Ganoderma lucidum*, had Brain-Derived Neurotrophic Factor-like neuronal-survival-promoting activities. Some monoterpenoids show beneficial effects on the cardiovascular system. Hemiterpenoids exhibit, among other activities, hepatoprotective effects. Carotenoids, which are also triterpenoids and have already been mentioned in this article, display a number of important protective effects in human health, including antioxidant, anti-inflammatory, and anti-cancer properties, the inhibition of malignant tumor growth, anti-aging, anti-atherosclerosis, and the induction of apoptosis [242].

Several essential oil compounds exhibit antioxidant activity. For instance, chamazulene from *Matricaria chamomilla* reduced ROS in endothelial cells, while terpenoids such as α-pinene, limonene, and linalool demonstrated radical scavenging effects, with α-pinene showing particularly strong activity. Additionally, α-terpineol, c-cadinene, geraniol, linalool, citronellol, cinnamaldehyde, eugenol, and isoeugenol, found in various essential oils also possess significant antioxidant capabilities [243]. Limonene, the main component of *C. sinensis* essential oil, exhibits moderate antioxidant and significant anti-inflammatory effects by scavenging free radicals and inhibiting pro-inflammatory responses in cells. Additionally, limonene and the essential oil show selective anticancer potential. It also exhibits promising antityrosinase and antielastase properties, suggesting benefits for skin health by potentially reducing hyperpigmentation and preserving elasticity [232].

**Figure 5.**
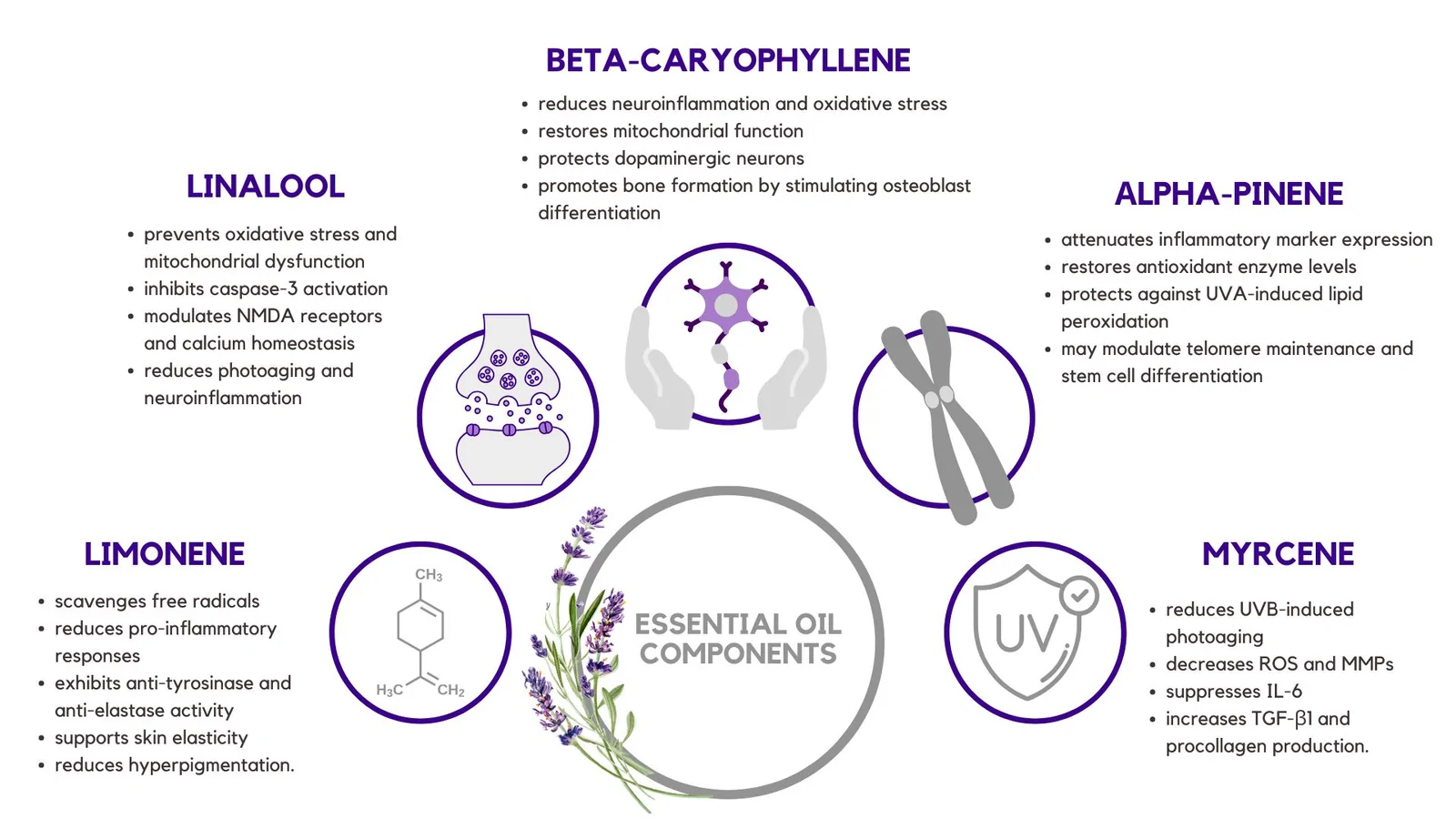
Examples of essential oils with anti-aging potential and leading mechanisms of their action [232-239].

Coriander essential oil demonstrates neuroprotective benefits by exhibiting *in vitro* antioxidant activity through scavenging hydrogen peroxide and reducing SOD activity. This could potentially prevent DNA cleavage induced by amyloid-beta oligomers. Lavender essential oil also shows neuroprotective effects against amyloid-beta-induced neurotoxicity. It reduces the activation of caspase-3 and the production of intracellular ROS. Linalool is a major component of both coriander and lavender essential oils. It has been shown that linalool can counteract mitochondrial dysfunction, reducing ROS levels. It has also been proven that, possibly through interaction with NMDA receptors and modulation of calcium homeostasis, linalool can inhibit caspase-3 activation [235]. Linalool prevents oxidative stress, inflammation, and photoaging in adult human dermal fibroblast cells [233]. Linalool inhibits inflammation both *in vitro* and *in vivo* and may be a potential therapeutic candidate for the treatment of inflammatory diseases [234]. β-Caryophyllene (BCP), a major sesquiterpene, has been reported to reduce neuroinflammation in models of Parkinson’s and Alzheimer’s disease by inhibiting inflammatory mediators and microglial activation. In PD models, BCP has shown neuroprotective effects. It reduces oxidative stress, restores mitochondrial function, and protects dopaminergic neurons. In the case of another age-related disease- osteoporosis, BCP also promotes bone formation by stimulating osteoblast differentiation and inhibiting bone resorption by suppressing osteoclastogenesis [244]. BCP's ability to combat oxidative stress and inflammation, key drivers of aging and various diseases, suggests a potential role in promoting healthier aging [236]. Among other essential oil components, myrcene, alpha-bisabolol, α-pinene, and β-cyclocitral are worth mentioning. It has been reported that myrcene may protect against UVB-induced skin photo-aging by acting as an effective antioxidant, decreasing ROS and MMP production, reducing IL-6, and increasing TGF-1 and procollagen secretion in UVB-irradiated skin cells. Myrcene also exhibits anti-inflammatory activity [239]. Alpha-bisabolol combats aging and degenerative diseases through its potent antioxidant and anti-inflammatory actions, which protect the nervous and cardiovascular systems. In NDs like PD and AD, alpha-bisabolol demonstrates neuroprotective effects by reducing oxidative stress, inhibiting neuroinflammation, preventing neuronal degradation, and interfering with amyloid-beta aggregation and acetylcholinesterase activity [245]. α-pinene, on the other hand, demonstrates anti-inflammatory effects by attenuating inflammatory marker expression in myocardial infarction, reducing neuroinflammation in ischemic stroke, and inhibiting inflammatory pathways in macrophages. Furthermore, α-pinene exhibits antioxidant properties by restoring antioxidant enzyme activity, reducing lipid peroxidation in ischemic brains, and preventing UVA-induced lipid peroxidation in skin. α-pinene odor stimulation is linked to health span, suggesting a potential anti-aging effect [237]. Studies indicate that alpha-pinene plays a role in delaying or limiting the differentiation of embryonic stem cells, potentially by influencing telomere maintenance [238]. β-cyclocitral (CYC), isolated from *Lavandula angustifolia Mill*, has been reported in preclinical studies to activate telomerase and prolong telomere length in yeast and mammalian cells. Additionally, CYC combats oxidative and nitrosative stress and enhances autophagy. This could be contributing to its lifespan-extending effects [246].

Some bioactive compounds of Thyme *(Thymus vulgaris L.)*, such as ursolic acid, betulinic acid, and oleanolic acid, exhibit neuroprotective abilities. They inhibit glutaminase, which is relevant to the prevention and treatment of NDs. Additionally, linalool and geraniol in thyme essential oil possess anti-inflammatory activity against neuroinflammation, further suggesting neuroprotective effects [247]. Antioxidants present in thyme essential oil (TEO), mainly monoterpenes, monoterpene alcohols, and phenol derivatives such as thymol (48.96%) and p-cymene (29.15%), are suggested to protect telomeres from oxidative damage associated with aging. This telomere protection and the anti-inflammatory activities of monoterpenes such as thymol and p-cymene suggest that dietary TEO supplementation can promote healthy aging. A study by Warman et al. explored the potential effects of TEO on age-related brain inflammation and telomere attrition. TEO, which was previously reported for its potency against neuroinflammation, also showed a notable impact on inflammation in the hippocampus and cerebellum of chronologically aged C57BL/6J male mice. Previous studies have indicated that the telomere shortening rate may be different not only between species but also between individuals, owing to genetic factors [247].

It has been reported that some components of essential oils, like β-cyclocitral, activate telomerase and lengthen telomeres in mammalian cells [249]. Autophagy-enhancing and antioxidant pathways may be involved in this effect. Neuroprotection in Parkinson's and Alzheimer's disease models, bone preservation through β-caryophyllene, and skin photoprotection through linalool and myrcene are examples of tissue-specific evidence [238, 239, 242, 247, 249]. But there is currently no data on direct binding to telomerase. Although several essential oil components show anti-aging potential and some have been studied in relation to telomere maintenance, most act indirectly by reducing oxidative stress. For many compounds, the precise mechanisms remain unclear, effects may vary between individuals, and effective concentrations or dosages have not been well established.

### 2.6 Bioactive postbiotics

Postbiotics have been defined by the International Scientific Association of Probiotics and Prebiotics (ISAPP, 2021) as ‘a preparation of inanimate microorganisms and/or their components that confers a health benefit on the host,’ a definition now widely adopted in the field [248]. Postbiotics are functional foods that may have promising health effects [249]. Direct studies on the impact of defined postbiotics on telomere length remain scarce; however, available evidence suggests they may indirectly influence telomere dynamics through modulation of oxidative stress and inflammation. Postbiotics may have a special effect on gut-organ axes and particular senescence pathways in addition to the general oxidative stress-reducing and inflammation-modulating effects previously mentioned. They also hinder cellular aging by influencing pathways such as mTOR signaling and impaired autophagy [250]. Future research specifically designed to investigate this connection would be valuable.

Several postbiotic metabolites have been shown to alleviate senescence-associated phenotypes by downregulating cell cycle inhibitors and reducing secretion of senescence-associated inflammatory factors, in addition to their general antioxidant and anti-inflammatory actions [251]. Heat-inactivated postbiotics improved intestinal barrier integrity and stimulated regeneration of intestinal stem cells in animal and in vitro studies, suggesting potential to counteract age-related gut alterations [252]. Recent studies highlight a gut-vessel axis linking microbiota to cardiovascular function. Postbiotics may exert cardioprotective effects via both local intestinal actions and systemic vascular signaling, although human evidence remains limited [253]. Postbiotics demonstrate a wide-ranging impact on the cellular mechanisms underlying NDs. This occurs through direct modulation of central nervous system cell signaling pathways or indirect influence on brain function through the gut-brain axis [250].

Topical application of EPI-7-derived postbiotics has been reported to enhance skin barrier function and improve elasticity and dermal density, suggesting a role in defending against photoaging [254]. Topical application of a *Streptococcus thermophilus* cream can increase ceramide levels in the stratum corneum. LactoSporin, derived from *Bacillus coagulans*, can provide antioxidant protection against UV damage, inhibit enzymes that break down collagen and elastin, and boost the production of key skin components. Lysates from *Bacillus amyloliquefaciens* and *Lacti-caseibacillus paracasei* also protect skin from aging by activating antioxidant mechanisms. They influence signaling pathways, reducing matrix degradation, and inhibiting pigmentation. A postbiotic from *Micrococcus luteus* can mitigate collagen breakdown from UVB, promote collagen synthesis, and accelerate wound healing [255].

Although postbiotics have no direct telomerase binding data, they may indirectly affect telomere dynamics by lowering oxidative and inflammatory damage through their modulation of mTOR and NF-κB signaling [253-254]. Documented tissue-specific effects include improved skin elasticity, cardiovascular support through the gut-vessel axis, and neuroprotection via gut-brain interactions [256-258]. Future research should clarify specific mechanisms and validate anti-aging potential in controlled human studies.

## 3. Summary

Natural compounds such as flavonoids, carotenoids, peptides, polysaccharides, essential oils, and postbiotics have been investigated for their potential to modulate aging-related pathways, including telomere maintenance, inflammation, and mitochondrial function. Our analysis reveals that the main converging pathways among various natural compounds are telomere maintenance, inflammation modulation, and mitochondrial function, with supporting roles for other mechanisms. Natural substances with neuroprotective, metabolic, and anti-inflammatory properties include polyphenolic flavonoids like luteolin, kaempferol, and quercetin. Carotenoids and essential oils likewise show efficacy in maintaining telomere integrity and cellular homeostasis. At the same time, mitochondrial-derived peptides such as humanin and MOTS-c contribute to improved stress resilience and cognitive function. Additionally, bioactive poly-saccharides and postbiotics exert protective roles in systemic aging and tissue regeneration through epigenetic and metabolic modulation. Collectively, these findings suggest that natural compounds may contribute to aging intervention strategies by targeting multiple biological processes. However, their capacity to preserve cellular function and extend healthspan remains to be confirmed in rigorous clinical studies. Although mechanistic insights are encouraging, there is still limited adaptation into clinical practice. There are currently insufficient large-scale, carefully monitored human trials with clinically meaningful endpoints and standardized preparations. Before these compounds can be safely suggested for use in prevention or treatment, future research should address issues of formulation, bioavailability, and long-term safety.

## Data Availability

Data sharing is not applicable to this article as no new data were created or analysed in this study.
